# La miliaire hépatique: une présentation échographique rare de la tuberculose hépatique découverte chez un adolescent immuno-compétent

**Published:** 2011-05-09

**Authors:** Kouamé N'goran, Evelyne Akaffou, Anhum Nicaise Konan, Anne-Marie N'goan-Domoua

**Affiliations:** 1 Service de radiologie, CHU de Yopougon, BP 632 Abidjan, Côte d'Ivoire; 2 Service de Pédiatrie-Néonatologie, CHU de Yopougon, BP 632, Abidjan, Côte d'Ivoire

**Keywords:** Echographie hépatique, miliaire hépatique, tuberculose extra-pulmonaire, tuberculose hépatique

## Abstract

Ce cas clinique rapporte un cas de tuberculose hépatique de présentation échographique exceptionnelle. Nous avons réalisé l’échographie abdominale d'un adolescent de 12 ans, immuno-compétent, sans notion de contage tuberculeux, se plaignant de douleur abdominale chronique évoluant depuis 8 mois associée à des signes frustres d‘imprégnation tuberculeuse. L'examen échographique a été réalisé à l'aide d'un appareil écho-doppler de marque Logic 400 de la firme General Electric. L’échographie abdominale a mis en évidence, une hépatomégalie comportant de multiples micro-abcès hypoéchogènes homogènes, mesurant entre 3 et 4 mm de diamètre et disséminés dans tout le parenchyme hépatique donnant l'aspect de miliaire. Une ascite de moyenne abondance localisée dans le pelvis a été objectivée. Il n'y avait pas d'adénopathies profondes ni de nodules péritonéaux. La ponction biopsie hépatique sous guidage échographique a permis de faire le diagnostic de tuberculose hépatique à l'histologie et à la bactériologie. Le patient a été mis sous traitement spécifique avec une surveillance échographique mensuelle. La disparition des micro-abcès et le tarissement de l'ascite ont été obtenus au bout du 4^ème^ mois du traitement. Celle de l'hépatomégalie est survenue un mois plus tard. L’échographie joue un rôle très important dans la détection précoce de la tuberculose hépatique, son diagnostic précis et la surveillance du traitement. Lors de sa réalisation dans un contexte de douleur abdominale chronique chez l'enfant, l’échographiste devrait évoquer le diagnostic de tuberculose hépatique devant une hépatomégalie multi-micro-abcédée, même chez l'enfant immuno-compétent.

## Introduction

La tuberculose hépatique se définit comme une localisation hépatique du bacille de Koch (*Mycobacterium tuberculosis*) qui est un bacille alcoolo-acido-résistant (BAAR). Elle se rencontre de façon isolée ou dans le cadre d'une atteinte multi viscérale [1]. Elle représente une entité rare de la tuberculose extra-pulmonaire [2, 3] surtout chez le sujet immuno-compétent [4]. Son incidence est estimée à 3% des tuberculoses extra-pulmonaire et 9% des tuberculoses abdominales [1]. Son traitement ne se dissocie guère des autres formes de tuberculose [4, 5]. Son évolution sous traitement reste favorable avec une régression en quelques mois des signes radiologiques [6] et histologiques [7, 8]. Mais elle pose un véritable problème diagnostic, même en zone d'endémie, à cause de son caractère insidieux et de l'aspect non spécifique de ses signes cliniques [1, 5, 9]. L'imagerie joue un rôle capital dans le diagnostic de la tuberculose hépatique [10, 11]. Elle permet sa découverte de façon fortuite ou dans le cadre de l'exploration d'une douleur chronique de l'hypochondre droit. Cette imagerie, le plus souvent représentée par l’échographie abdominale [5], peut s'aider des nouvelles techniques [3, 6, 12, 13] telles que la tomodensitométrie (TDM) ou l'imagerie par résonance magnétique (IRM). Elle permet surtout de poser le diagnostic de certitude qui est histologique ou bactériologique en guidant la ponction biopsie du foie [6].

Nous rapportons un cas de tuberculose hépatique, chez un enfant de 12 mois, immuno-compétent, présentant une douleur abdominale chronique, chez qui l'examen échographique a permis le diagnostic et la surveillance. Notre but étant d'attirer l'attention sur une présentation échographique particulière de cette affection : la miliaire hépatique.

## Observation

**Figure 1:**
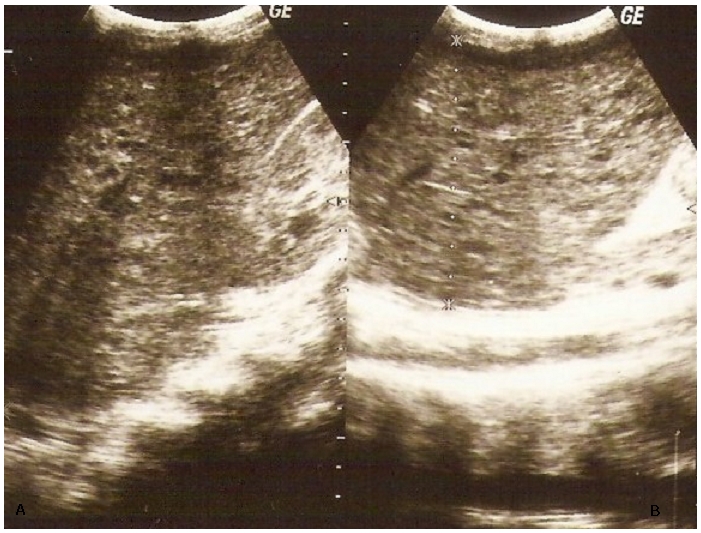
Echographie du foie en coupe longitudinale parallèle à l'axe du rein droit à l'aide d'une sonde de basse fréquence (3,5 Mhz) montrant le lobe droit (A) et le lobe gauche (B) du foie chez un enfant de 12 ans. Le foie est hypertrophique avec un parenchyme truffé de micronodules hypoéchogènes: tuberculose hépatique.

**Figure 2:**
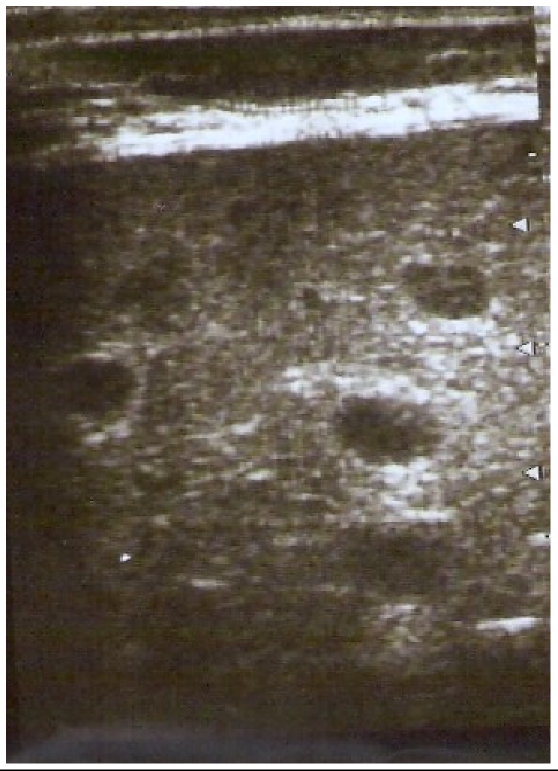
Même patient. A l’échographie abdominale à l'aide d'une sonde de haute fréquence (7,5 Mhz), les micronodules et l'ascite pelvienne sont mieux visualisés.

L'enfant KKL, adolescent de 12 ans se plaignait de douleur abdominale chronique évoluant depuis 8 mois sans localisation précise. Il était suivi et traité comme colopathie fonctionnelle dans un hôpital périphérique sans succès. Il n'y avait pas de notion de contage tuberculeux. La sérologie rétrovirale était négative. Il présentait des signes frustres d'imprégnation tuberculeuse avec une altération de l’état général, une douleur de l'hypochondre droit et un ballonnement abdominal d'apparition récente. L'examen clinique conduit par le pédiatre a mis en évidence une hépatomégalie et un météorisme abdominal. Il n'y avait pas d'ictère. Le bilan biologique a objectivé une élévation de la CRP à 48 mg/l, sans hyperleucocytose et des phosphatases alcalines à 180UI/l. L'intradermo-réaction à la tuberculine était positive (phlycténulaire). Une échographie abdominale, une radiographie de l'abdomen sans préparation et une radiographie thoracique de face ont été réalisées. Les images radiographiques de l'abdomen sans préparation et du thorax étaient normales. L'examen échographique a été réalisé à l'aide d'un appareil écho-doppler de la firme General Electric muni de 2 sondes dont l'une superficielle (7,5 Mhz) et l'autre profonde (3,5 Mhz). Il nous a permis d'objectiver une hépatomégalie multi micronodulaire disséminée (Figure 1 et 2) dans tout le parenchyme hépatique et une ascite de moyenne abondance localisée dans le pelvis (Figure 2). Les micronodules étaient hypoéchogènes homogènes sans signal doppler. Ils mesuraient entre 3 et 4 mm de diamètre. Leurs parois étaient hyperéchogènes épaisses et peu régulière. Ils présentaient parfois des renforcements acoustiques postérieurs (Figure 2). Nous n'avons pas retrouvé d'adénopathies profondes ni de nodules péritonéaux. Le diagnostic d'hépatomégalie multi-micro-abcédée a été évoqué à l’échographie. Une ponction biopsie hépatique sous guidage échographique a été réalisée. L'examen histologique a mis en évidence un granulome giganto-épithélio-cellulaire avec nécrose caséeuse centrale. La culture sur milieu spécialisé a mis en évidence des BAAR. Le patient a été mis sous traitement antituberculeux avec une surveillance échographique mensuelle. Cette surveillance échographique du traitement a mis évidence une disparition des nodules et un tarissement de l'ascite au bout du 4^ème^ mois du traitement. La disparition de l'hépatomégalie est survenue un mois plus tard.

## Discussion

Chez le sujet immunocompétent, en dehors d'une localisation multi-viscérale, la tuberculose hépatique est exceptionnelle. Chez l'enfant, la tuberculose hépatique sévit le plus souvent aux alentours de 12 ans [2, 5]. Elle est caractérisée par l'absence de signes spécifiques et son diagnostic est souvent orienté par des douleurs chroniques de l'hypochondre droit [1]. Les signes d'imprégnation tuberculeuse peuvent manquer. Dans notre observation, ces signes sont survenus tardivement. Sur le plan biologique, le syndrome inflammatoire est présent chez 50 % des malades et une cholestase anictérique peut être associée [14]. Chez notre patient, le syndrome inflammatoire était limité à une élévation de la C-Réactive protéine. Il n'y avait pas d'ictère et les phosphatases alcalines étaient normales pour l’âge. La radiographie thoracique de face peut dans le cadre d'une tuberculose multi viscérale être contributive en objectivant une localisation parenchymateuse pulmonaire ou une atteinte pleurale [9]. Mais dans la tuberculose hépatique isolée, seulement l'ascite peut être retrouvée. D'o[ugrave] l'importance de l’échographie qui réunit à la fois les capacités de pouvoir mettre en évidence l'ascite, rechercher des ganglions et des nodules péritonéaux (dans le cadre d'une tuberculose abdominale) mais surtout de pouvoir explorer le foie. Dans notre observation, l'ascite a été retrouvée à l’échographie. Elle n'avait aucun caractère pouvant orienter vers la tuberculose hormis sa localisation pelvienne. Le foie était hypertrophique. L'hépatomégalie, retrouvée déjà à l'examen clinique fait partie des signes indirects de l'atteinte hépatique tuberculeuse. Elle est constante [1]. Les autres signes d'atteinte hépatique tuberculeuse sont variables à l’échographie. Il peut s'agir d'une simple stéatose [4], d'une fibrose [7] ou d'une masse tumorale [8, 12]. D'autres fois l'atteinte tuberculeuse hépatique fait discuter à l’échographie un abcès amibien ou à pyogène [1, 3]. Mais la forme la plus fréquemment observée est la forme nodulaire [12]. Ces nodules [3] pouvant être de grande taille (tuberculose macronodulaire) ou alors de petite taille (forme micronodulaire). Cette dernière forme est celle que nous avons rencontrée. Il s'agissait de micronodules hypoéchogènes diffusément repartis dans tout le parenchyme hépatique et donnant l'aspect de miliaire tuberculeuse telle qu'elle est décrite dans sa localisation pulmonaire. Ces nodules étaient des micro-abcès dont certains étaient collectés et d'autres non. D'o[ugrave] l'inconstance dans notre observation du renforcement acoustique postérieur. Le diagnostic de tuberculose n'est pas clinique ni imagerique. Il est histologique (recherche du granulome tuberculeux avec nécrose caséeuse centrale) ou bactériologique (recherche de BAAR). Pour cela, devant la suspicion échographique de tuberculose hépatique, en présence de la positivité de l'intradermo-réaction à la tuberculine, nous n'avons pas jugé nécessaire de demander d'autres techniques d'imagerie (TDM et IRM) comme certains auteurs l'ont fait [1, 10, 12–14]. Nous avons effectué, sous guidage échographique, une ponction biopsie hépatique avec étude histologique à la recherche d'un granulome avec nécrose caséeuse centrale et mise en culture du matériel pour la recherche bactériologique des BAAR [1]. Ceci a permis de confirmer le diagnostic avec pour corollaire une mise en route rapide d'un traitement spécifique adapté et efficace ayant permis une guérison avec disparition des micronodules et tarissement de l'ascite avant la fin du traitement.

## Conclusion

La tuberculose hépatique est rare et pose un problème diagnostic chez l'enfant immuno-compétent. L’échographie aide à son diagnostic précoce et participe à tous les points de sa prise en charge. L’échographie abdominale est peu couteuse, disponible et non irradiante. Lors de sa réalisation dans un contexte de douleur abdominale chronique chez l'enfant, l’échographiste devrait évoquer le diagnostic de tuberculose hépatique devant une hépatomégalie multi-micro-abcédée, même chez l'enfant immuno-compétent. Cette présentation échographique particulière de la tuberculose hépatique sous la forme d'une miliaire hépatique ou d'une hépatomégalie multi-micro-abcédée mérite d’être divulguée.
